# Cost-effective track and trace technology for poor-quality chemotherapeutic pharmaceuticals in resource-limited countries: a review of the Chemotherapeutic Paper Analytical Device

**DOI:** 10.3389/fmedt.2024.1436614

**Published:** 2024-11-21

**Authors:** Minichil Chanie Worku, Liknew Workie Limenh, Biset Asrade Mekonnen, Yeniewa Kerie Anagaw

**Affiliations:** ^1^ Department of Pharmaceutical Chemistry, School of Pharmacy, College of Medicine and Health Sciences, University of Gondar, Gondar, Ethiopia; ^2^ Department of Pharmaceutics, School of Pharmacy, College of Medicine and Health Sciences, University of Gondar, Gondar, Ethiopia; ^3^ Department of Pharmacy, College of Medicine and Health Sciences, Bahir Dar University, Bahir Dar, Ethiopia

**Keywords:** quality, cancer, substandard, counterfeited, chemotherapeutic product, ChemoPAD, cost-effective

## Abstract

Poor-quality medicines (substandard or counterfeit) can lead to treatment failure. There is a vast global imbalance in cancer treatment outcomes due to the difficulty of accessing quality chemotherapeutic products. Early diagnosis of cancer brings more hope for curative treatment of cancer and increases the demand for chemotherapeutic products. Consequently, it creates opportunities for unethical manufacturers and suppliers to develop substandard and/or counterfeit products. An ongoing review of cost-effective analytical methods is therefore paramount to tracking and tracing poor chemotherapeutic pharmaceutical products. Low- and middle-income country (LMIC) regulators lack safety equipment and standard operating procedures to handle chemotherapeutic products safely in the drug analysis laboratory and have limited capacity to perform post-marketing surveillance on these products. This review aimed to provide a compressive review of the Chemotherapeutic Paper Analytical Device (ChemoPAD). ChemoPAD is an important tool for quality screening of commonly used chemotherapeutic products in LMIC settings. It is an efficient, fast, simple, accessible, cost-effective, and transferable analytical method for verifying substandard and/or counterfeit chemotherapeutic products. Designed as a complete paper-based laboratory the size of a playing card, the ChemoPAD provides a promising solution for healthcare providers, patients, and other parties involved in post-marketing surveillance of chemotherapeutic products. Thus, in the near future, scientists could probably witness the use of the ChemoPAD technology platform to trace and track substandard and/or counterfeit chemotherapeutic products.

## Introduction

High-quality drugs are required for effective illness management, as poor-quality medicines (substandard or counterfeit) can lead to treatment failure and severe responses, complicating the disease. The pharmaceutical industry has developed a vast range of new, specific pharmaceutical products and has evolved in the sophistication of finished product manufacturing, thus contributing to raising health standards in most countries (1). However, parallel to this, the circulation of toxic, substandard, and counterfeit drug products in national and international markets has increased (2). The quality of medicines can readily deteriorate through improper handling, distribution, or storage before reaching consumers. Therefore, quality control in the distribution system, per proper specifications, is a vital prerequisite for ensuring optimal treatment outcomes (2, 3).

The pharmaceutical plant possesses peculiar characteristics that distinguish it from popular perceptions. It is a field filled with uncertainty, sometimes manufacturing poor-quality pharmaceutical products (1). Pharmaceutical products should be controlled to protect public health and to ensure that medicinal products in the national and international markets are safe, effective, high-quality, and produced following good manufacturing practices (2). Quality screening and evaluation at different outlets are thus vital for ensuring the quality of medicines. It provides information on handling, storage, and manufacturing conditions that can affect the quality of products.

Quality screening and evaluation of chemotherapeutic products should hence be a routine activity for pharmaceutical analysts. In fact, a vast global imbalance exists in cancer treatment outcomes, owing to factors such as difficulty in accessing treatment with chemotherapeutic products (4). Pharmaceutical products, including chemotherapeutics, are essential for public health and should typically be available and accessible to the public (2). Cancer treatment mostly leads to fateful costs for clients and their families, which exacerbates the existing imbalance (4). These impacts are aggravated by infiltrating poor-quality chemotherapeutic products into healthcare settings. A combination of market forces, low per capita spending on medicinal products by most of the population, and limited resources for quality control and monitoring creates an environment favorable for introducing substandard and/or counterfeit chemotherapeutic products in low- and middle-income countries (LMICs) (2).

Accordingly, an updated review on cost-effective analytical methods for post-marketing surveillance of chemotherapeutic products is essential to track and trace poor chemotherapeutic pharmaceutical products. The review includes a brief overview of cancer prevalence, the quality of chemotherapeutic products, and analytical technologies for tracking and tracing substandard and counterfeit chemotherapeutic products.

### Cancer treatment synopsis

Cancer is a major non-communicable disease (NCD) (5, 6) characterized by the uncontrolled proliferation of abnormal cells in the body, which can infiltrate normal body tissues. Many cancers and the aberrant cells that compose the cancer tissue are named according to the tissue or organ from which they originated (for instance, breast cancer, lung cancer, acute leukemias, thyroid cancer, and colorectal cancer) (7).

Effective treatment of cancer relies on access to effective chemotherapeutic products (4). For various types of cancer, different types of chemotherapy medications can be recommended: alkylating agents (cisplatin, oxaliplatin), antimetabolites (methotrexate), anthracyclines (doxorubicin), etc. Each group of drugs and/or their combinations targets the affected cells through different mechanisms (7).

Providing safe and affordable access to chemotherapeutic products is a significant challenge in the care of patients with cancer, especially in LMICs (4, 8). Cancer is a major contributor to the global disease burden (5). While communicable diseases remain the leading causes of death in many LMICs, the incidence and mortality rates of NCDs (including cancer) are rising rapidly. This has resulted in a twofold disease burden, which is imposing strain on the existing healthcare system (6).

The number of cancer patients is constantly increasing, leading to an increase in the number of different chemotherapy treatments administered (5, 9). Early diagnosis brings more hope for curative treatment and increases the demand for chemotherapeutic products that are often in limited supply (4, 5, 8, 10). This increase in demand for chemotherapeutic products may create opportunities for unethical suppliers and illicit smugglers to doorway substandard and/or counterfeit chemotherapeutic products at the outlets.

## Quality control of chemotherapeutic drugs

### Quality of pharmaceuticals

The global pharmaceutical market is characterized by a footing of multiple standards of pharmaceutical product quality. Many National Medicine Regulatory Authorities (NMRAs) in LMICs lack the capacity to properly guarantee the quality of the products circulating within their boarders, leaving the most vulnerable populations at risk of receiving poor-quality pharmaceutical products (11, 12). Hence, globalization can potentially spread poor-quality pharmaceutical products, substandard and counterfeit, before adequate detection and intervention are possible (13).

Substandard drugs are those that fail to meet specification ranges cited in the pharmacopeia or in the manufacturer's approved dossier. “They, out of specification products, are genuine medicines produced by manufacturers, authorized by the NMRA, which fail to meet quality specifications set for them by national standards” (13–15).

Any pharmaceutical product being manufactured anywhere in the world can be imitated by others. In some occasions, imitated pharmaceutical products are sold under the brand name of the original manufacturer. Such products are called counterfeit, fake, falsified, phony, or bogus (13). There is no clear, universally agreed-upon definition for counterfeit medicines (CFMs). The most widely used definition in the literature is that given in 1992 by the WHO. This defines CFMs as medicines that are “deliberately and fraudulently mislabeled with respect to identity and/or source” (15). Counterfeiting can occur with both branded and generic products. CFMs may contain the correct ingredients, incorrect ingredients, no active ingredients, insufficient ingredients, or fake packaging (15, 16).

The scale of pharmaceutical product trafficking differs significantly from country to country. LMICs in Africa and southeast Asia (China and India) are prime target markets (17). Poorer countries are preyed on more compared to developed counterparts. Substandard drugs and CFMs make a complex and critical global health issue (18). The WHO has received many reports of substandard and/or counterfeit pharmaceutical products, including chemotherapeutic drugs (15).

### Quality of chemotherapeutic pharmaceutical products

Up to 2 billion people worldwide lack access to essential medicines, creating a vacuum that is often filled with substandard and/or counterfeit products (15). This problem is growing as global drug supply chains become more complex (12). Although the presence of many pharmaceutical plants and distribution channels for pharmaceutical products can improve the healthcare system, unfortunately, the ineffective regulation of pharmaceutical production and trade has significantly increased the circulation of substandard and/or counterfeit products (2, 4, 19).

It is estimated that about 10% of pharmaceutical products found in LMICs, including chemotherapeutic drugs, are either substandard or counterfeit (15, 17, 20). These products are ongoing issues in LMICs and especially in sub-Saharan Africa, where they comprise 34% of the market (14, 19). This implies that they are major threats to patients and constitute a public health menace. Some articles report even higher figures (21).

The ill turn taken by substandard and/or counterfeit chemotherapeutic products in LMICs is hard to determine against the backdrop of high rates of morbidity and mortality, as many patients are diagnosed at advanced stages and face insufficient access to these products (8). Cancer research brings hope for a cure with adjuvant chemotherapy, which further increases the demand (8, 10). In fact, chemotherapeutic products are already in limited supply in LMICs.

Access to quality-assured chemotherapeutic products is integral to tackling disparities in cancer treatment outcomes and fostering a global patient-centered public health approach (4). In line with this, six sub-Saharan countries have partnered with the American Cancer Society (ACS) and the Clinton Health Access Initiative (CHAI) to improve access to 16 key chemotherapeutic products, including anastrozole, bleomycin, capecitabine, carboplatin, cisplatin, cytarabine, docetaxel, doxorubicin, epirubicin, fluorouracil, gemcitabine, leucovorin, methotrexate, oxaliplatin, paclitaxel, and vinblastine (22). Increased access to these chemotherapeutic products will create opportunities for unethical manufacturers and suppliers, a pattern seen in other global health interventions.

Chemotherapeutic products are particularly attractive targets for falsification due to their high selling prices (4, 8, 23) and because greedy providers know they are unlikely to be caught. Many regulatory bodies in LMIC lack safety equipment and standard operating procedures to handle chemotherapeutic products safely in drug analysis laboratories and have limited capacity to perform post-marketing surveillance on these products (10, 23). Although pharmacopeial assays exist for almost all drugs, chemotherapeutic products present unique challenges for drug inspectors and laboratory analysts in LMICs due to their high toxicity and the limited infrastructure setup (23).

It is critical to ensure that patients can obtain and afford pharmaceutical products through formal and quality-assured channels, but this needs adequate coverage for chemotherapeutic products. These products are routinely excluded from national healthcare plans, primarily due to their high cost. LMICs often pay 20–30 times more for generic medicines, highlighting the need to strengthen acquisition through pooled procurement initiatives (4).

There is insufficient published information about the prevalence of substandard or falsified chemotherapeutic products. The Pharmaceutical Security Institute (PSI) ranks these products as the fifth most commonly counterfeited drug class (23). A particularly serious case involved counterfeit versions of bevacizumab (Avastin), a chemotherapeutic product. Avastin's manufacturer, Roche, notified (physicians in February 2012) that a counterfeit version of bevacizumab, containing only fillers (salt and starch), was circulating (24). Moreover, a study conducted at Tikur Anbessa Specialized Hospital (TASH), Ethiopia, between 1 and 14 September 2018, tested 20 vials of Cisteen (cisplatin) samples and identified them as substandard. Chemotherapeutic Paper Analytical Device (ChemoPAD) results indicated that some lots of a particular cisplatin product were suspect. Confirmatory testing by high-performance liquid chromatography (HPLC) showed that this product contained 54% ± 8% of the stated active ingredient, confirming that it was a substandard product (23). The purpose of this review was to critically appraise the ChemoPAD technology for tracking and tracing substandard and/or counterfeit chemotherapeutic products circulating in LMIC markets.

## Methods

This study aimed to ascertain the importance of ChemoPAD technology. A literature search was carried out using the following medical databases: EMBASE, Google Scholar, ScienceDirect, MEDLINE, PubMed, the PSI, and International Pharmaceutical Abstracts. Moreover, preliminary search with MeSH terms from related published articles was conducted to choose the most specific and sensitive words for the search strategy. Specific areas, such as cancer and chemotherapeutic drugs, in relation to quality track and trace methods with ChemoPAD, were recognized and included as additional terms to increase sensitivity; however, the search was not restricted solely to these categories. The search used MeSH terms like fake, counterfeit, substandard or falsified, and poor quality combined with drugs, medicines, pharmaceuticals, anticancer, and antineoplastic. In addition to the articles, different guidelines and related books were also investigated.

### Track and trace analytical technology for chemotherapeutic products

As a serious public health problem, substandard and/or counterfeit pharmaceutical products demand urgent intervention. To counteract these products, many intervention measures, such as strong regulation and monitoring, must be utilized to ensure quality across the supply chain (2, 25). Substandard products and CFMs are significant problems in many LMICs, where technological infrastructures are inadequate to detect these harmful products (10).

Poor health outcomes can erode trust in the pharmaceutical industry, even for genuine products. Differentiating substandard products or CFMs is often challenging without verification technologies (18, 26). Hence, there is a need for efficient, fast, simple, accessible, and transferable analytical methods that can be used for the detection and analysis of substandard and/or counterfeit chemotherapeutic products (10).

Various screening methods could be used to perform verification of pharmaceutical products, including Raman spectroscopy (RS), infrared (IR) spectroscopy, ultraviolet–visible (UV–vis) spectroscopy, thin-layer chromatography (TLC), high-performance thin-layer chromatography (HPTLC), and HPLC to mention some (27). However, these instruments are rarely available or affordable in LMICs (2, 10).

Commercially available instruments for field screening of pharmaceutical products in LMICs include the Global Pharma Health Fund (GPHF) Mini-Lab, developed by Merck (Darmstadt, Germany) (15, 23). The estimated cost of a single GPHF Mini-Lab is around €700–900 (28). However, there is currently no affordable verification technology for detecting substandard and/or counterfeit chemotherapeutic products at the point of use (10).

While LMICs have several GPHF Mini-Labs at multiple customs sites, this tool is not an option still for field screening of chemotherapeutic products because it uses flammable solvents and does not include chemotherapeutic agents in its drug list (10). Currently, the global number of clients receiving chemotherapeutic products has increased considerably (29). Given the toxicity of cytotoxic products to patients, the development and application of cheap, accessible, and reliable analytical methods to screen and analyze these products have become necessary. Among all the analytical methods, the paper analytical device (PAD) stands out for fulfilling the criteria mentioned for determining the most commonly used chemotherapeutic products in LMIC settings (10, 23). These paper-based tests have pioneered the development of new analytical tools for on-site pharmaceutical product analysis (30).

### Paper analytical device

Paper has been utilized in chemical measurements for centuries, dating back to the use of litmus paper to measure the power of hydrogen (pH) in the 1700s (30). While paper has been used as a substrate for chemical analysis for centuries, the concept of microfluidic PADs was only recently introduced by Whitesides et al. (30, 31). Paper is a hydrophilic material; hence, its affinity with water allows solutions to flow through its porous structure. This simple capillary action does not require additional mechanical construction for pumping (30).

PAD represents a breakthrough technology for rapid field screening of pharmaceutical products in LMICs (23, 32). Unlike litmus paper, PADs use chemical printing and/or cutting to define flow channels, making it possible to conduct multifold analysis using small sample volumes (30). PADs are designed as a complete laboratory on a playing-card-sized paper.

PAD has been employed for various applications, including environmental, pharmaceutical, and forensic sciences, as well as in the food and beverage industries. It is a fast and inexpensive analytical method (30, 32, 33). PADs are designed to perform rapid field screening and determine the presence of adulterants in pharmaceutical products in a qualitative and semi-quantitative manner (32).

PADs can be used at many points in the drug supply chain by both governmental and non-governmental organizations to detect low-quality pharmaceutical products [active pharmaceutical ingredient (API) < 90%], which pose significant health risks (20, 32). For instance, PADs can screen some antibiotics with high infectious burdens in LMICs, such as amoxicillin, azithromycin, ceftriaxone, ciprofloxacin, and doxycycline. Some common antimalarial drugs, such as chloroquine and hydroxychloroquine, are also included. Furthermore, combinations of the four first-line anti-tuberculosis (TB) drugs, namely, rifampicin, isoniazid, ethambutol, and pyrazinamide, were developed to detect adulteration (20).

The growing popularity of PADs is primarily due to their inherent advantages, including requiring small sample and reagent volumes, generating minimal waste, low cost, small weight and size, and simple point-of-care (POC) sensor designs (23, 30, 34). PADs have attracted attention as a new POC diagnostic platform due to their handiness and design, especially in LMICs (33). PADs enable the creation of sensors that can be made quickly. The colorimetric application of PADs qualitatively estimates the concentration of adulterants based on the color produced in the assays (31, 33).

Fabrication of analytical devices based on microfluidic structures and lab-on-a-chip platforms has advanced dramatically (33). PADs can be fabricated using any type of porous membrane with the right combination of thickness, pore distribution, price, and absorption rate (30). Although numerous grades of paper exist, only a few are used to make PADs, with Whatman paper achieving a golden standard status in the field (33). Physical techniques can be used to plug pores in paper substrates vertically, and photolithography is used to design devices that require a copier machine or an inkjet printer, ultraviolet (UV) light, and a hotplate (30, 31).

Patterning the hydrophilic membrane creates hydrophobic barriers so that the analytes and reagents can flow to specific regions upstream on the PAD. Various patterning methods, such as photolithography, printing (wax), cutting, and chemical vapor deposition, are used to define hydrophobic barriers (30). Wax printing has been popular for PAD fabrication because of its significant benefits, including low cost, simple fabrication, high speed, robustness, and the absence of pollutant organic solvent consumption. It forms hydrophobic barriers and hydrophilic channels by printing wax patterns on the paper surface (30). According to Scida et al., some studies report breakthrough PAD designs that provide timed reactions, simple assembly by folding the paper substrate, and non-enzymatic signal amplification (35). These all represent significant advances because they provide important functionalities without significantly increasing device complexity.

### Chemotherapeutic Paper Analytical Device

Various modern analytical methods are used to analyze chemotherapeutic products, but they are very expensive, non-portable, require well-trained personnel, and sometimes need derivatization. In addition, many national laboratories lack the setup for these products. Nowadays, a breakthrough analytical method, named ChemoPAD, has emerged to address such problems due to its aforementioned advantageous (35). According to Smith et al., it is possible to develop a quality control system for chemotherapeutic products that allows caregivers (ward personnel) to test the quality of chemotherapeutic products while the patients is receiving treatment (10).

In addition to the absence of access to the latest instruments, LMICs face significant shortages of high-purity chemicals, solvents, and supplies needed to perform golden standard quality testing. Reputable suppliers may not deliver to these areas, or delivery could take months, leaving health professionals in a dilemma (23). POC testing uses experimental analyses designed to deliver quick results directly at the site of patient care. These tests can be carried out by personnel with minimal training in principles of working in low-resource settings. ChemoPAD meets the eight criteria for POC tests, namely, ASSURED (affordable, sensitive, specific, user-friendly, rapid and robust, equipment-free, and delivered to those in need) (33).

PADs provide breakthrough solutions to logistical problems (33). Hence, ChemoPAD is an important option for screening chemotherapeutic products, especially in LMIC settings where instrumental methods are neither available nor affordable. Against the high background of cancer patient mortality, the absence of drug side effects might be the only clue for health practitioners that a chemotherapeutic product is substandard or counterfeit. This study changed this situation by enabling quick, on-site screening of these products (10).

**Figure 1:**
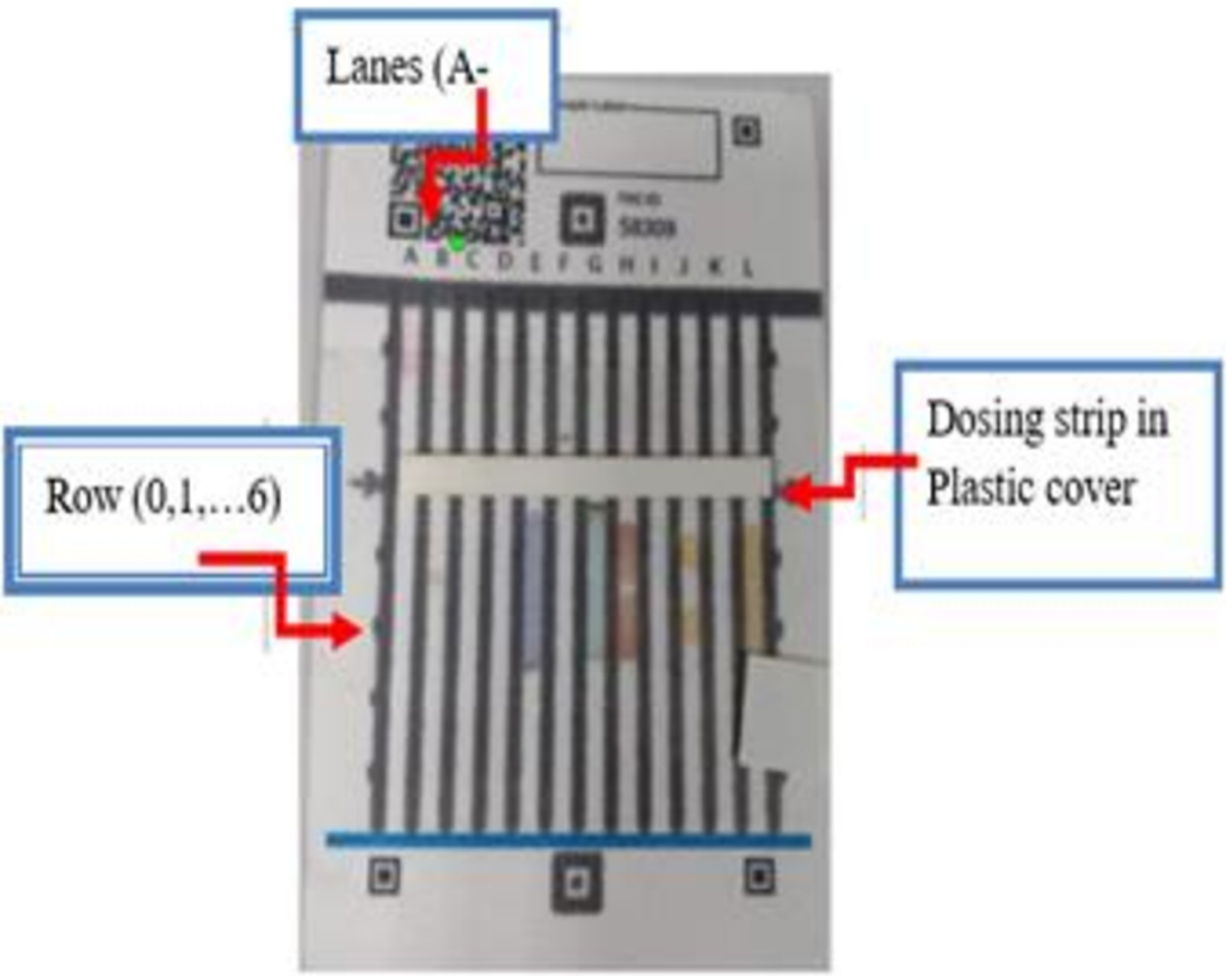
ChemoPAD pre-dosed with dried reagent.

ChemoPAD was developed collaboratively at the University of Notre Dame (UND) and Addis Ababa University (AAU), TASH, to screen the quality of four commonly prescribed chemotherapeutic products in LMICs, namely, cisplatin, oxaliplatin, doxorubicin, and methotrexate (10). It is also possible to fabricate this device locally anywhere. ChemoPAD contains 12 lanes (A–L), each pre-dosed with color reagents, which are stored in dry form (Figure 1).

ChemoPADs were produced using wax printing on Ahlstrom paper (10) to create separate reaction areas. Small amounts of reagents are deposited to create unique color barcodes in response to different APIs in the chemotherapeutic product. The dimension is 7 cm × 11 cm.

The ChemoPAD can simultaneously run a dozen chemical color tests on a chemotherapeutic product sample within a few minutes. It tests the drug, not the packaging, and each ChemoPAD costs a few dollars (around $1/ChemoPAD) to fabricate (Figure 1).

**Figure 2:**
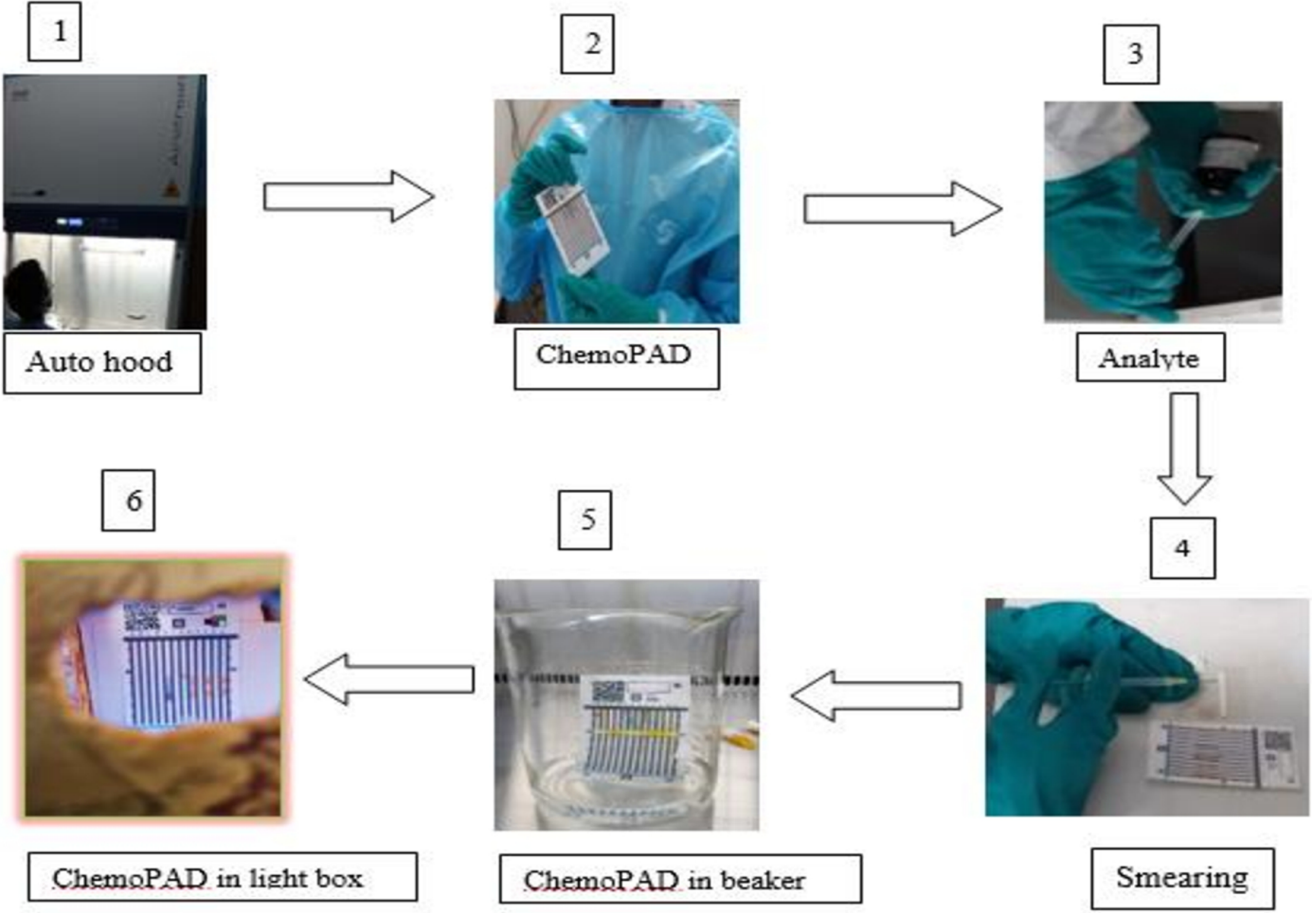
Schematic of ChemoPAD screening: (1) switch on the chemo fume hood and wait for 3 min; (2) take off ChemoPAD, pre-dosed with dried reagents from aluminum foil sac (zip-top bag) and make it ready; (3) aspirate around 60 µl of the injectable analyte with an insulin syringe from the ampule; (4) smear the injection on the dosing paper strip and fold over the adsorbent paper immediately; (5) immerse it in a baker containing water at the base; and (6) dry the card at ambient temperature and screenshoot image in the lightbox.

The ChemoPAD has a plastic cover with an absorbent paper strip onto which an injectable chemotherapeutic analyte is placed. The card is then folded, which deposits small spots of the analyte from the absorbent paper strip in all 12 lanes. The bottom edge of the card is then placed in water, which draws up all components on the lanes (A–L) by capillary action. That means the water dissolves the color reagents stored in the lanes and sweeps them over the drug spots (Figure 2). The resulting unique color barcodes reveal the presence of functional groups in the chemotherapeutic product, and the color intensities can allow for the semi-quantification of certain chemotherapeutic products (10, 20).

The PAD was developed as a cost-effective tool for field screening a wide variety of pharmaceutical products in dosage form in low healthcare infrastructure settings (10, 32). As a preliminary test method, it shares a concept similar to TLC to identify substandard and/or counterfeit products. For instance, a case study in Kenya showed that using PAD to screen substandard and counterfeit amoxicillin can save an average of $9,100 over 3 years compared to relying solely on HPLC assays (36).

A study conducted in Blantyre urban townships, Malawi, used the PAD to screen 42 amoxicillin samples, none of which contained suspicious products (37). Another screening using the PAD in Kenya identified many lots of amoxicillin and doxycycline adulterated with talcum powder, counterfeit paracetamol, and substandard losartan (38) very low cost.

The ChemoPAD is a simple and fast test for assessing the quality of chemotherapeutic products, requiring only a small beaker of water and an insulin syringe to apply the injectable analyte. This test was developed and validated to detect the presence of four APIs, namely, cisplatin, oxaliplatin, doxorubicin, and methotrexate, at the POC (10).

Limited published data are available on the screening of ChemoPADs. A study conducted at TASH, Ethiopia, found substandard Cisteen (generic name: cisplatin) while screening with the ChemoPAD (23). Moreover, quality screenings of parenteral chemotherapeutic drugs (cisplatin, oxaliplatin, doxorubicin, and methotrexate) were conducted on samples collected from the University of Gondar Compressive Specialized Hospital (UoGCSH), northwest Ethiopia and from Addis Ababa, central Ethiopia. Even though no defects were seen during visual inspection, the ChemoPAD screening results ascertained the presence of substandard cisplatin (brands Cisother, Namanaplatin, and Platinox), methotrexate, and doxorubicin (brands Adrosal and Robol) products, with a very minimum cost at the outlets (39).

### Future perspective of ChemoPADs

This review studies the architecture of a ChemoPAD system to track and trace substandard and counterfeit chemotherapeutic products and also predicts future trends in the method's application.

Researchers have constantly explored technologies (40) that improve the day-to-day lives of human beings, including developing low-cost, paper-based microfluidic devices and exploring new applications by incorporating efficient detection methods (40, 41).

ChemoPAD is a promising technology that, if efficiently implemented into the drug supply chain cycle, could bring numerous benefits, including healthcare providers, patients, and other parties involved in the post-marketing surveillance services (10, 20, 23). However, several challenges remain that must be addressed before the ChemoPAD can be ubiquitously deployed in healthcare services. Basically, PAD technology has certain limitations, including sample retention within paper fluidic channels and evaporation during transport, resulting in the low efficiency of sample delivery; also, some hydrophobic agents used for patterning cannot build hydrophobic barriers strong enough to withstand samples of low surface tension, and the limit of detection (LOD) is usually high in colorimetric methods integrated into PADs, making them insufficient for the analysis of samples with very low concentrations (40). The same challenges are true for the ChemoPAD (20, 40).

The innovation in ChemoPAD technology is still in its early stages; significant research efforts will be needed to develop and validate more chemotherapeutic drugs in this field to include and nurture it into a more mature platform technology. Further exploratory studies will be conducted to discover new concepts and capabilities of this technology (40). While individual researchers in this field may come up with a list of potential future directions from their point of view, here, we hope to convey to readers a few of the perspective directions that we think are relevant and attractive in this field. Thus, in the near future, scientists could probably witness using ChemoPAD technology platforms to trace and track substandard and/or counterfeit chemotherapeutic products.

### Limitations

This review has limitations concerning the searching strategies for the different studies used since there was no pre-specified and structured questionnaire. Also, there is a scarcity of literature on post-marketing surveillance studies on chemotherapeutic pharmaceutical products. Although the reviewers will gain knowledge about the issue, they may not have a thorough grasp of the current state of the science.

## Conclusion

This review studies the ChemoPAD system to track and trace substandard and counterfeit chemotherapeutic products. In LMICs, the increase in demand for chemotherapeutic products creates opportunities for unethical suppliers and illicit smugglers to doorway substandard and/or counterfeit products at the outlets. ChemoPAD is thus an ideal analytical method that can be used for field screening these substandard and/or counterfeit chemotherapeutic products. ChemoPAD innovation is still in its early stages; significant research efforts will be needed to develop and validate more chemotherapeutic drug items in this field.
